# Advance Care Planning and Care Coordination for People With Parkinson's Disease and Their Family Caregivers—Study Protocol for a Multicentre, Randomized Controlled Trial

**DOI:** 10.3389/fneur.2021.673893

**Published:** 2021-08-05

**Authors:** Marjan J. Meinders, Giovanni Gentile, Anette E. Schrag, Spiros Konitsiotis, Carsten Eggers, Pille Taba, Stefan Lorenzl, Per Odin, Kristina Rosqvist, K. Ray Chaudhuri, Angelo Antonini, Bastiaan R. Bloem, Marieke M. Groot

**Affiliations:** ^1^Scientific Center for Quality of Healthcare, Radboud Institute for Health Sciences, Radboud University Medical Center, Nijmegen, Netherlands; ^2^Department of Neurology, Center of Expertise for Parkinson and Movement Disorders, Donders Institute for Brain, Cognition and Behavior, Radboud University Medical Center, Nijmegen, Netherlands; ^3^Department of Neuroscience, University of Padua, Padua, Italy; ^4^Department of Clinical and Movement Neurosciences, UCL Institute of Neurology, University College London, London, United Kingdom; ^5^Department of Neurology, Medical School, University of Ioannina, Ioannina, Greece; ^6^Department of Neurology, Philipps University Marburg, Marburg, Germany; ^7^Knappschaftskrankenhaus Bottrop GmbH, Department of Neurology, Bottrop, Germany; ^8^Department of Neurology and Neurosurgery, Institute of Clinical Medicine, University of Tartu, Tartu, Estonia; ^9^Neurology Clinic, Tartu University Hospital, Tartu, Estonia; ^10^Institute of Nursing Science and Practice, Paracelsus Medical University, Salzburg, Austria; ^11^Department of Neurology and Department of Palliative Care, Ludwig-Maximilians-University, Munich, Germany; ^12^Department of Neurology, Klinikum Agatharied, Hausham, Germany; ^13^Division of Neurology, Department of Clinical Sciences Lund, Lund University, Lund, Sweden; ^14^Department of Basic and Clinical Neuroscience, Parkinson's Foundation Centre of Excellence, King's College London, London, United Kingdom; ^15^Department of Anesthesiology, Pain and Palliative Care, Radboud University Medical Center, Nijmegen, Netherlands

**Keywords:** Parkinson's disease, palliative care, advance care planning, care coordination, family caregiver

## Abstract

**Background:** Parkinson's disease (PD) is a progressive neurodegenerative disease with motor- and non-motor symptoms. When the disease progresses, symptom burden increases. Consequently, additional care demands develop, the complexity of treatment increases, and the patient's quality of life is progressively threatened. To address these challenges, there is growing awareness of the potential benefits of palliative care for people with PD. This includes communication about end-of-life issues, such as Advance Care Planning (ACP), which helps to elicit patient's needs and preferences on issues related to future treatment and care. In this study, we will assess the impact and feasibility of a nurse-led palliative care intervention for people with PD across diverse European care settings.

**Methods:** The intervention will be evaluated in a multicentre, open-label randomized controlled trial, with a parallel group design in seven European countries (Austria, Estonia, Germany, Greece, Italy, Sweden and United Kingdom). The “PD_Pal intervention” comprises (1) several consultations with a trained nurse who will perform ACP conversations and support care coordination and (2) use of a patient-directed “Parkinson Support Plan-workbook”. The primary endpoint is defined as the percentage of participants with documented ACP-decisions assessed at 6 months after baseline (t1). Secondary endpoints include patients' and family caregivers' quality of life, perceived care coordination, patients' symptom burden, and cost-effectiveness. In parallel, we will perform a process evaluation, to understand the feasibility of the intervention. Assessments are scheduled at baseline (t0), 6 months (t1), and 12 months (t2). Statistical analysis will be performed by means of Mantel–Haenszel methods and multilevel logistic regression models, correcting for multiple testing.

**Discussion:** This study will contribute to the current knowledge gap on the application of palliative care interventions for people with Parkinson's disease aimed at ameliorating quality of life and managing end-of-life perspectives. Studying the impact and feasibility of the intervention in seven European countries, each with their own cultural and organisational characteristics, will allow us to create a broad perspective on palliative care interventions for people with Parkinson's disease across settings.

**Clinical Trial Registration:**www.trialregister.nl, NL8180.

## Introduction

Parkinson's disease (PD) is the second most common neurodegenerative disease worldwide, affecting 1–2% of the world population above 65 years of age. The number of people with PD is expected to double from 6.9 million in 2015 to 14.2 million in 2040 (1). On average, people live for 15 years with the disease (2–4). As the disease progresses, people develop a range of motor as well as non-motor symptoms, which typically increase over time. For example, in a European cohort of 692 people diagnosed with late-stage PD and an average disease duration of 15 years, 68% reported off-periods for at least 50% of the day, 82% reported falls, and 92% experienced at least one neuropsychiatric symptom, with apathy, depression, and anxiety most commonly being present (5, 6). Furthermore, around 60% of patients with PD will ultimately develop dementia (7, 8). In light of this complex and multifaceted phenotype, it is understandable that treatment programs are complex, that quality of life becomes progressively threatened, and that informal carers experience considerable distress. However, despite the very high symptom burden at the end of life, end-of-life care in the field of PD often is not aligned with patients' needs and preferences (9, 10). Palliative care is often not introduced: in a cohort of advanced PD patients in Germany, with a mean disease duration of 17 years, 72% of the participants expressed an unmet need for palliative care (11). A large study including ~125,000 people with PD showed that 43% died in a hospital and only 9.7% in their homes, which is substantially lower compared to the 17% of the general elderly population dying at home. Hospice services were barely utilized, that is, in only 0.6% of the patients (12).

To address these challenges, there is growing awareness of the potential benefits of palliative care for people with PD (13, 14). According to the World Health Organization definition, published in 2012, palliative care is “an approach that improves the quality of life of patients and their families facing the problems associated with life threatening illness, through the prevention and relief of suffering by means of early identification and impeccable assessment and treatment of pain and other problems, physical, psychosocial and spiritual” (15). Advance Care Planning (ACP) is a cornerstone for palliative care, involving the timely identification and definition of goals as well as preferences for future medical treatment and care, discussion of these goals and preferences with family and healthcare providers, and recording and reviewing of these preferences if appropriate (16). There is a vast amount of international evidence, particularly in the field of oncology, on the benefits of palliative care in improving quality of life, increasing satisfaction with care and, for some patients, prolonging life (17–19).

Although the importance of palliative care for chronic neurological conditions has been well-established in the setting of clinical studies (13, 20, 21), in real life, many PD patients do not receive the support they need. Unlike conditions that are life-threatening immediately after diagnosis, the sense of urgency seems to be lacking in a slowly progressive and long-lasting condition like PD. The unpredictable prognosis makes it difficult to define a clear referral cutoff point, which prevents neurologists from appropriately referring patients to specialist palliative care services. Moreover, many physicians lack the communication skills and do not want to take away hope and patience (22). PD patients' acceptance of their symptoms as part of their everyday life, believing that no effective treatments are available, is an important barrier to report non-motor symptoms (23), hampering the recognition of palliative care needs.

Only recently, several studies have explored how palliative care principles should be designed and implemented to effectively support and treat people with PD (24–28). Foremost, effective palliative care requires an individualized approach, and patients should actively be invited to discuss ACP early on in the course of the disease and on a regular basis; palliative care requires skilled professionals who are knowledgeable on both PD and palliative care, and ACP decisions should be clearly documented and shared with relevant services. Finally, given the multidisciplinary nature of palliative care, care coordination should be an explicit responsibility of the care team (24, 29, 30).

Further studies are needed to evaluate the positive effects of palliative care models across a range of healthcare systems. The current PD_Pal trial was designed to understand the impact of palliative care services for PD, within a wide range of European healthcare systems. An evidence-based intervention will be evaluated consisting of a nurse-led, person-centred palliative care model for people with PD living at home, assisted living situation, or nursing homes. The intervention deals with two major challenges that many people with PD encounter (31, 32):

1. Increasing risk of cognitive and/or communication impairments that hinder the ability to easily discuss or indicate preferences about healthcare and quality of life when the disease advances. Therefore, timely documentation of patients' wishes related to advanced and end-of-life care is essential, but rarely part of standard care.
2. The lack of care coordination during the transition from clinic-based care (focused on adjusting patients' medical treatment to control symptoms) to community-based care (focused on adjusting patients' care and daily living routines to comfortably live with the symptoms that can no longer be completely controlled).

The objective of this study is two-fold. First, we will determine the effectiveness of a nurse-led, person-centred palliative care intervention for people with PD and their family caregivers compared to care as usual. To evaluate this intervention, we will primarily focus on ACP documentation in the medical files, to demonstrate that relevant end-of-life issues were indeed discussed. Additional outcomes will focus on patients' clinical outcomes, caregivers' quality of life, patients' and caregivers' costs and service utilisation. Second, we will assess the feasibility of the PD_Pal intervention across seven European countries (Austria, Estonia, Germany, Greece, Italy, Sweden and United Kingdom).

## Methods and Analysis

### Study Design

**Figure 1:**
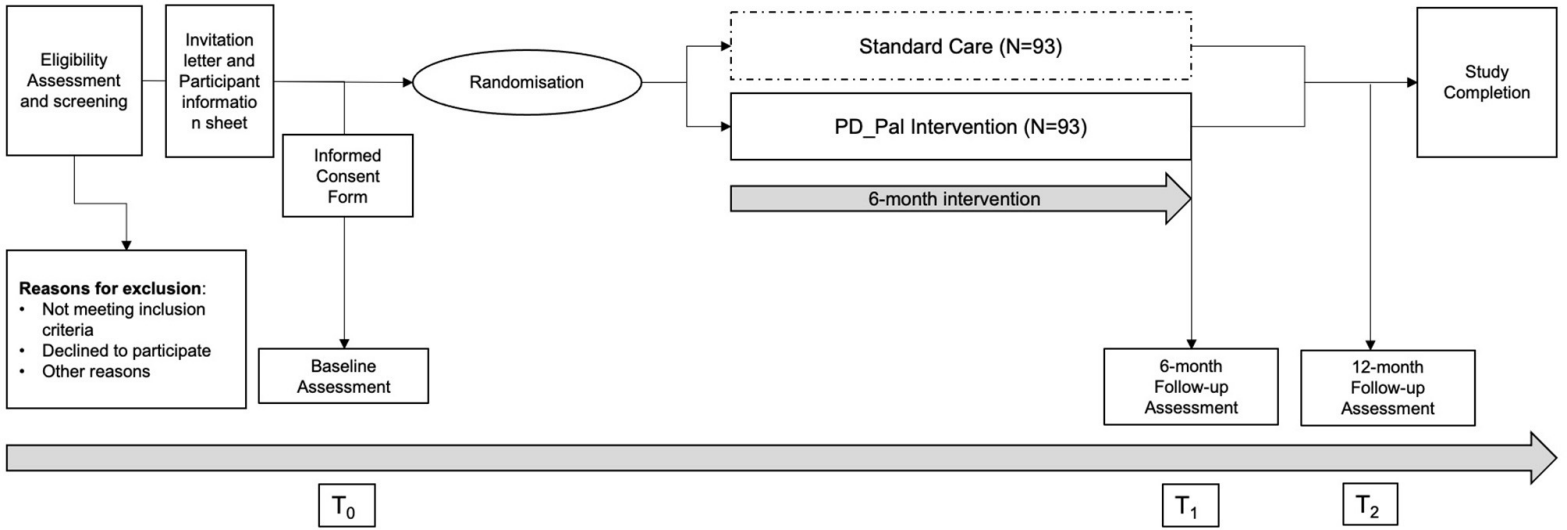
Study flowchart of the PD_Pal clinical trial.

The intervention will be evaluated in a multicentre, single-blinded randomized controlled parallel group design, in seven European countries. Within each participating country, one trial centre will lead the recruitment. Participants will be randomized in a 1:1 ratio to either the intervention or the control group, who will receive care as usual. The intervention will be delivered during the first 6 months after randomisation. Assessments will be performed at baseline (t0), at the end of the intervention phase, that is, after 6 months (t1), and after 12 months (t2) for follow-up (see Figure 1).

Participating clinical centres should have at least one movement disorder specialist available. Centres are excluded if they already apply a palliative care model as part of their routine care workflow; if they have detailed palliative care guidelines available with corresponding high-quality practices; and/or if the centre is participating or has participated in a palliative care study in the past 3 years.

### Study Population

The intervention targets individuals diagnosed with idiopathic PD or an atypical parkinsonism syndrome, independent of their age.

In order to be eligible to participate in this study, a person should meet all of the following criteria:

1. Meeting the clinical diagnostic criteria for PD, as defined by the Movement Disorders Society (33), or the criteria for an atypical parkinsonism syndrome (34);
2. Hoehn & Yahr ≥3 (35);
3. Progressive deterioration in physical and/or cognitive function despite optimal therapy, according to the primary physician;
4. Cognitively able to complete questionnaires and to participate in interviews;
5. Ability to provide written informed consent; and
6. Availability of a family caregiver or informal caregiver, jointly abbreviated as “FC” in the remainder of this article.

Furthermore, persons are excluded from participation if one of the following criteria are met:

1. Inability to communicate independently, with or without supportive communication tools;
2. Unable or unwilling to commit to study procedures;
3. Presence of additional chronic medical illnesses which may require palliative services (e.g., metastatic cancer);
4. Already receiving palliative care or hospice services; and/or
5. Already participating in a clinical study for palliative care.

Having a device-assistant advanced treatment [including deep brain stimulation (DBS), levodopa–carbidopa intestinal gel (LCIG), and continuous subcutaneous apomorphine infusion (CSAI)], or considering one, is not an exclusion criterion. We will identify patients who certainly have complex medical needs and at the same time are still able to commit to ACP conversations and make decision.

The participation of a FC is compulsory. The FC should meet the following criteria:

1. Willing to provide written informed consent;
2. Cognitively able to complete questionnaires and to participate in interviews;
3. Aging ≥ 18 years; and
4. Identified by the person with PD as the FC.

### Sample Size Calculation

We assume that 5% of the target population will have documented ACP wishes at baseline. The study is powered to show a 20% absolute increase from a baseline of 5% (control group) in the primary outcome measure, that is, documented ACP-decisions, at 6 months, with a power of 0.80, a statistical significance of 0.05 (two sided) and an intraclass correlation coefficient (correcting for clustering within countries) of 0.10, by a Fisher's exact test. With the above assumptions, 74 patients in each treatment group are required. The sample size will be increased to 93 patients in each treatment group for allowing a 25% dropout rate within the 6-month follow-up period for the primary outcome effectiveness evaluation.

### PD_Pal Intervention

The proposed PD_Pal intervention is the result of a systematic approach, where we first explored the views of healthcare professionals and patients on palliative care in the Netherlands (25, 36, 37). Subsequently, these findings were translated into the intervention: trained nurses and a workbook for patients. The specifically trained nurses, labelled as PD_Pal nurses in this study (see below), will be coordinating transmural, integrated, and proactive palliative care, including ACP, through regular conversations with patients and their FC. The conversations are supported with a patient-directed “Parkinson Support Plan—workbook,” designed to be used at home by the patient and FC to document their wishes and preferences related to end-of-life care, to prepare and guide the conversations with the PD_Pal nurse. The plan is structured within four steps (Table 1). These steps are based on previous theories [e.g., ACP (16)], shared-decision making (38), The Chronic Care Model (39), and empirical studies [e.g., interventions guiding ACP conversations that describe healthcare models or interventions aimed to provide care aligned with patients' needs, values, and preferences on all domains of palliative care, for example, physical, social, psychological, spiritual, and financial (40–43)]. The initial workbook was reviewed by a panel of five Dutch patients and caregivers and subsequently adapted based on their feedback. To make the workbook suitable for the international study, the PD_Pal nurse training started with a critical review of the workbook, and adapted to the national situation, where needed. Given the comprehensive scope of the workbook, the intervention goes beyond the clinical management of PD consequences.

**Table 1:** Defined steps in the Parkinson support plan.

**Step**	**Aim**
1—Individual care plan	Describe current health and caregivers, and identification of current needs related to care and care coordination.
2—Proactive care plan	Identify expected future challenges and care needs per domain (e.g., physical, social, psychological, spiritual, and financial). The leading theme in this step is: “What is needed for good care, now and in the future?” There is also attention for challenges and needs, as experienced by the family caregiver.
3—Quality of life and end-of-life plan	Identify and document the patient's ideas about quality of life, and preferences related to end-of-life care (e.g., surrogate decision maker; life prolonging procedures; and hospital or nursing home admissions).
4—Coordination and revision plan	Discuss and plan how the “Parkinson Support Plan” will be coordinated and reviewed in the future (e.g., contact (newly assigned if not already assigned) care coordinator, update him/her about the plan, and facilitate him/her in consulting a Parkinson expert when necessary; decide when and how the “Parkinson Support Plan” will be reviewed). The PD_Pal nurse and the dyad will allocate the referred to the assigned care coordinator, to guarantee continuity of the integrated palliative care beyond the PD_Pal study.

#### The PD_Pal Nurse

The PD_Pal nurses are trained to assist the participating patients in taking the four steps of the Parkinson Support Plan. The training consists of (1) face-to-face sessions to develop skills necessary to assist the patient during the intervention, including skills to deal with emotions, and (2) monthly digital coaching sessions with the intervention-coordinator (MMG) where experiences can be discussed. Nurses will be selected based on the following criteria:

1. Previous experience in nursing (preferably on a “Bachelor of Nursing” or comparable level);
2. Experienced in delivering care for people with Parkinson's disease and/or atypical parkinsonism syndromes OR experienced in delivering palliative care;
3. Being able to visit patients at home/at a clinical centre;
4. Able to speak and write in English (training will be held in English);
5. Being able or willing to talk about the end of life; and
6. Open attitude toward (differences in) patients' preferences and values in life.

The delivery of the intervention (e.g., setting, timing, frequency, and content) will be tailored as much as possible to the patient's and FC's preferences and possibilities. Although the duration of the total intervention is tailored to patients' preferences, the study design and timeline mandate the following limits: the first conversation with the PD_Pal nurse should be scheduled up to 4 weeks after randomisation and the last conversation up to 6 months after the randomisation.

#### The Control Group

Patients in the control arm receive care-as-usual from their neurology and/or home care team. The care-as-usual and the extent to which ACP is part of this care are expected to differ among the participating countries.

### Recruitment and Consent

Several methods to reach the target group are employed, building upon the experiences with patient recruitment in the Care for Late Stage Parkinsonism (CLaSP) study (5, 44). First of all, the participating neurology clinics recruit participants from their outpatient and inpatient clinics and registries of patients who have indicated to be interested in research participation. Neurology clinics can only act as a recruitment centre if they do not offer palliative care services themselves. Second, the study centres will contact geriatricians, general practitioners, nursing homes, patient advocate groups, and self-help groups to draw attention to the project and identify and recruit eligible patients. Identified clinicians give written information to patients about the study, and if patients are interested and willing, the clinician completes a standard referral form and sends it to the local research team. The research team will contact the patient by phone, explain the trial, check the eligibility criteria as far as possible in a phone call, and will send the full information package. Patients will have at least 1 week to consider participation. If a patient provides verbal consent to contact their FC, the research team will approach the FC and invite the FC for participation as well, following the same procedure as outlined for the patient.

Patients who are interested in participation, meet the selection criteria, and have provided their initial, verbal consent will be given a first appointment for a screening visit with a study assessor, which could be a physician (neurologist, geriatrician, or psychiatrist), study nurse, or trained researcher. During the screening visit, information about the study will be explained again, and if the participant still agrees, the informed consent form will be signed. Subsequently, eligibility criteria will be verified in the screening visit, before collecting any baseline clinical and demographic data. In case the eligibility criteria can be verified based on a telephone interview and review of the medical records, written informed consent will be obtained without a screening visit. All participants will be able to withdraw their informed consent to parts or to the overall participation at any point in time.

### Randomisation, Blinding, and Treatment Allocation

Participants are considered to be enrolled into the study following written informed consent, confirmation of eligibility, and allocation of the participant ID number. After inclusion, a patient will participate in the baseline assessment (t0), after which the patient will be randomized to either the intervention or the control group (1:1) by a computer-generated algorithm embedded within the certified eCRF system. A member of the research team will communicate to the patient and FC the group to which they have been assigned.

The trial is single-blinded, as patients and their FCs cannot be blinded for treatment allocation. Participants are urged not to discuss their allocation status with the blinded study assessor, who is responsible for the data collection. At each visit, the assessor will record to which study arm they think the participant was allocated, which will allow us to assess the efficacy of the blinding.

### Study Endpoints

#### Primary Endpoint

The primary endpoint is defined as the percentage of participants with documented ACP decisions in at least one of the patients' medical records assessed at 6 months (t1) after baseline. We believe it is important to choose an outcome measure that is as close as possible to the intervention. The choice of documentation of ACP decisions as the primary endpoint was prompted by a number of considerations. One of these is that even though discussing ACP is a crucial part of the intervention, such a discussion by itself does not ensure better care, and adequate documentation is therefore a further prerequisite. Another consideration is that this endpoint proved to be sensitive to change in similar interventions targeting other populations (40).

#### Secondary Endpoints

Secondary endpoints relate to the expectation that patients and their FCs will experience a better quality of life, improved care coordination, and a reduced patient symptom burden and that FC will experience an improved quality of life, in a cost-effective manner.

#### Other Endpoints

To characterize the population, we will collect demographic and social information from the participants. Furthermore, we will evaluate the feasibility aspects of the intervention and we will document what is needed to tailor the intervention procedures and materials to country-specific characteristics (e.g., differences in language and organisation of care).

In those participants enrolled in the trial who provide a separate consent, wearable sensor data will be collected, by using the PDMonitor system. The PDMonitor system consists of five devices which will be attached to both shanks and wrists and the lower back. Each device contains an accelerometer, a gyroscope, and a magnetometer. The PDMonitor is a CE-marked product, certified as Medical Device class IIa. The system has been validated for PD-related motor-symptoms, for example, bradykinesia, dyskinesia, tremor, freezing of gait, gait disturbances, postural instability, ON/OFF conditions, and response fluctuations (45, 46). In PD_Pal, data will be recorded during daily living after baseline (t0), and after each follow-up visit (t1 and t2), for five consecutive days (morning to evening) for a maximum of 12 h per day. The data will be used for further validation and exploratory analysis, for example to see if the data can serve as a predictor for the primary and secondary outcomes (e.g., how activity level and severity motor symptoms measured at home are related to the frequency of ACP arrangements, or patients' and caregivers' quality of life).

### Assessment Scheme

The baseline assessment (t0) consists of an in-person interview performed by the study assessor. The baseline assessment takes place either in the outpatient clinic setting, at the patient's home, or remotely via a video connection. In addition, the patient and FC complete a set of questionnaires that are self-administered. Within 2 weeks after the baseline assessment, a participant is randomized to either the intervention or the control group. For all participants, two follow-up assessments are thereafter foreseen (t1 and t2). After completion of the t2 assessment, the patient and FC will be invited for an optional semi-structured interview about end-of-life issues. Table 2 presents all assessments and the instruments that will be used to evaluate the (cost-)effectiveness and feasibility of the PD_Pal intervention.

**Table 2:** Overview of the assessment schedule and its instruments, to evaluate the (cost-) effectiveness and feasibility of the PD_Pal intervention.

**Scales/domains**	**Instruments**	**Application at**		
		**T0**	**T1**	**T2**
**Study rater completed, together with the patient**				
Demographics / social data		X		
Motor symptoms	MDS-UPDRS, part III (47)	X		
Non-motor symptoms	MDS-Non-Motor Rating Scale (MDS-NMS) (48)	X	X	X
Cognition	Montreal Cognitive Assessment (MoCA) (49)	X		
Comorbidity	Charlson Comorbidity Index (CCI) (50)	X		
Care coordination	Modified Nijmegen Continuity Questionnaire	X	X	X
	(mNCQ) (51)			
	Interview questions^***^	X	X	X
Feasibility of the intervention^**^	Feasibility checklist		X	
(Serious) adverse events	Interview questions		X	X
**Study rater completed, together with the FC**				
Demographics		X		
Resource utilisation	Resource Utilisation questionnaire (RUD) (52), adapted for PD	X	X	X
[if applicable] Quality of the end-of-life experience of the patient^*^	Quality of Dying and Death questionnaire (QoDD) (53)		X	X
**Questionnaires completed by the patient independently**				
Disease-specific symptoms	Edmonton Symptom Assessment Scale for Parkinson's Disease (ESAS-PD) (54)	X	X	
Depression	Beck Depression Inventory (BDI-I) (55)	X	X	
Quality of life	PDQ-39 (56)	X	X	X
Self-rated health	EQ-5D-5L (57)	X	X	X
Palliative-phase symptom severity	Integrated Palliative Care Outcome (IPOS) (58)	X	X	X
Experienced quality of care (including the intervention)	Short Assessment of Patient Satisfaction (SAPS) (59)	X	X	
Experienced involvement in decision making	CollaboRATE (60)	X	X	
**Questionnaires completed by the FC independently**				
Quality of life	EQ-5D-5L (57)	X	X	X
	PQoL Carer (61)	X	X	X
**Study rater competed**				
ACP documentation	Chart review	X	X	X
[if applicable] Place of death: preferred and actual	Chart review		X	X
**Interview with patient and FC**				
Feasibility of the intervention**/^***^	Interview guide		X	
Experienced quality of care, quality life, and end-of-life issues^***^	Interview guide			X
**Quantitative motor symptom assessment**				
Motor symptom assessment^***^	PDMonitor	X	X	X

* *in case the patient dies during follow-up;*

** *intervention group only;*

*** *Optional element of the study protocol; T0, Baseline after inclusion; T1, 6 months after randomisation (intervention completed); T2, 12 months after randomisation (long-term follow-up)*.

### Statistical Analysis

#### Primary Endpoint

To evaluate the effectiveness of the intervention, all analyses of endpoints will be done in the intent-to-treat population. The primary efficacy analysis will be to investigate the effect of the PD_Pal intervention on the percentage of patients with documented ACP decisions from baseline to month 6 in the intervention and care-as-usual group. This will be tested by means of the Mantel Haenszel estimate method and by using multilevel logistic regression model, with data clustered within countries and with categorical factors (groups) and baseline characteristics as covariates, in order to test which independent variables (indicators) contribute to the effect of the intervention. The primary analysis will be repeated for the t2 assessment (12 months after randomisation) and also using the per-protocol population to confirm the overall study results. All tests will be performed two-sided, and *P*-values < 0.05 will be considered statistically significant.

#### Secondary Endpoints

All secondary study parameters, except for the healthcare utilisation data, will be analysed similarly to the primary outcome parameter, except that for the secondary outcome measures we will correct for multiple testing (Bonferroni adjustments). For the healthcare utilisation data, the primary analysis will be from a health and social care cost perspective, with secondary analyses from a societal perspective.

#### Gender-Specific Analyses

We will undertake a planned subgroup analysis for the primary outcome measure, separately for women and men.

#### Other Study Parameters

Information regarding perceived care coordination and feasibility of the intervention will be obtained in optional qualitative interviews. To assure the quality of this multicentre, multinational, multi-language qualitative study, we will build on the lessons learned from the CLaSP trial (62), in which many of the study centres participated, and on recommendations and experiences described in the literature (63).

Software for qualitative analysis will facilitate data storage, coding, searching both within and across sites, and participant groups, retrieving data and recording analytical thinking (e.g., NVivo or AtlasTi). The data are linked with the quantitative data to interpret the change in patients/FCs of the quantitative outcome measures, their clinical significance, and the impact of the intervention at two levels (people and context; processes and tasks), and to identify ways to enhance the intervention and the processes for wider implementation. Quality appraisal is addressed through procedures to ensure systematic and rigorous attention to analysis and reporting.

### Data Management

Each investigator will document subject data in his/her own subject files. These subject files will serve as source data for the study. Data collected during this study as recorded on the appropriate source documents will be entered in a web-based electronic data capture (EDC) system specifically developed for the study and provided by the clinical research organisation (CRO) and project partner, Mediolanum Cardio Research (MCR), Milano, Italy. The e-CRFs will be reviewed periodically for completeness, consistency, and query status by the data management personnel of the CRO. Remote monitoring will be regularly performed by the CRO staff in order to oversee the progress of the study, completion, and quality of collected data. The raw sensor data, collected with the PDMonitor devices, and its processed data are uploaded and pseudonymized stored at the PD Neurotechnology's cloud platform.

### Harms

All adverse events, adverse reactions, and serious adverse events or reactions that occurred from the signature of the informed consent during the whole study duration will be recorded in the specific section of the e-CRF. Death events due to disease progression will not be considered as serious adverse event (SAE); however, data will also be recorded in a specific section of eCRF. Adverse events will be collected and coded using the most current version of the Medical Dictionary for Regulatory Activities (MedDRA). Each adverse event will be categorized by severity (mild, moderate, severe) and seriousness (serious, non-serious). The investigator will follow up the outcome of any Adverse Events (clinical signs, laboratory values or other, etc.) until the return to normal or consolidation of the patient's condition. In the case of any Serious Adverse Event, the patient will be followed up until clinical recovery is complete and laboratory results have returned to normal, or until progression has been stabilized.

## Discussion

The awareness of the possible merits of palliative care interventions for people with PD is growing (14, 64). However, we still do not fully understand how to optimally design palliative care models, and little is known about its potential impact for this patient population (13). In 2020, two large randomized controlled trials published the effects of multidisciplinary palliative care teams, with inconsistent results. The first one, conducted in American outpatient clinics, evaluated a multidisciplinary palliative care model for PD patients and their family caregivers. The patients received palliative care support in person or by telemedicine sessions, every 3 months for 12 months. The study showed a modest, but significant improvement in patients' quality of life after 6 months, leaving caregiver burden unchanged. In addition, non-motor symptom burden, motor symptom severity, completion of advance directives, caregiver anxiety, and caregiver burden favoured the intervention group at 12 months (24). The second study targeted people with long-term neurological conditions, including PD. This UK-based study evaluated a short-term palliative care intervention, using a comprehensive assessment, personalized care planning, case management, and care coordination, and advising existing care providers. The intervention lasted 6–8 weeks, with three distinct sessions for the patient and family caregiver with the multi-professional palliative care teams. After 12 weeks, no change in eight key palliative care symptoms emerged, although the intervention was associated with lower healthcare costs (65).

The PD_Pal intervention takes a different approach: instead of involvement of a multidisciplinary care team, we will assign a dedicated nurse, who will act as the personal case manager for the patient and family caregiver. The nurse will lead the conversations, create a relationship based on mutual trust, and involve other disciplines whenever needed. We deliberately opted for an intervention strategy, which combines specific training of a nurse in relevant areas of knowledge and skills with a prolonged in-depth support intervention for the patients and FCs. Throughout the intervention period, the nurses will join monthly digital meetings to share their experiences and discuss encountered problems and solutions. The Parkinson Support workbook is designed as a strategy to increase active engagement of patients and FCs in the ACP conversations about the future. Furthermore, the intervention is patient-centred and the patients will be deciding what will be discussed and when. None of the (sub)steps within the intervention are obligatory and patients can also add certain care (coordination) issues that are not included in the Parkinson Support Plan. To summarize, the PD_Pal model is advocating active engagement of patients as a key element for effective palliative care interventions (66).

The COVID-19 pandemic has affected the execution of the trial, which was planned to start in February 2020. The pandemic shows the importance of discussing goals of care, and to revisit or establish advance care plans in an early phase of the disease. Furthermore, the pandemic forced us to deploy telehealth solutions for e-consent, e-scales, and e-delivery of the intervention. The original protocol already included an option for teleconsultations for the PD_Pal nurses, as a measure to be inclusive for those patients who would live too far away from the clinical site for regular face-to-face visits. Now we anticipate to use a teleconsultation model as a necessary alternative, leveraging on earlier experiences. A review of 71 studies (67) concluded that, on the positive side, patients generally experience more comfort and control at home, leading to an exclusive digital connectedness between conversation partners. In contrast, professionals can experience reservations about addressing painful truths and emotional topics during teleconsultations as they did not feel sufficiently close. We will therefore strive for a first face-to-face contact between the PD_Pal nurse and the patient, before a teleconsultation solution will be applied. Nevertheless, the pandemic creates a unique opportunity to learn important lessons about the application of telehealth solutions in clinical research.

To conclude, studying the impact and feasibility of the intervention in seven European countries, each with their own cultural and organisational characteristics, will create a substantial body of knowledge about the future of palliative care for people with PD and their family caregivers.

## Ethical Considerations and Dissemination

The PD_Pal study design has been developed following the indications contained in the Charter of Fundamental Rights of the European Union. Informed consent will be obtained by each participant as in the Declaration of Helsinki (2013) and in line with ethic committee approval of the protocol. None of the steps within the intervention are obligatory. Patients can indicate they do (not yet) want to discuss or think about certain topics. All participants will be able to withdraw their informed consent to parts or to the overall participation at any point in time. When participants develop cognitive deficits, together with the FC, we will evaluate their ability and willingness to continue participating in the study.

The project results will be disseminated through the MDS Task Force on Palliative Care, the European Association for Palliative Care, the European Academy of Neurology, and the European Parkinson's disease association. In addition, dissemination will be accomplished through scientific publication on national and international journals as well as through participation to scientific and communication events related to the study topics. It is also important that the progress and findings are presented to PD patients and caregivers (usually in regional audiences) and by publishing lay summaries. PD_Pal will support the open-access (OA) initiative. OA literature is digital, online, free of charge, and free of most copyright and licensing restrictions. OA to research articles both in journals (“gold OA”) and in repositories (“green OA”) is foreseen.

## Ethics Statement

The studies involving human participants were reviewed and approved by the local ethics committees of all participating study sites (UK: London—Central Research Ethics Committee, REC reference 20/LO/0122, IRAS project ID 271717; Sweden: Etikprövningsmyndigheten, registration number 2020-00032; Austria: Ethikkommission für das Bundesland Salzburg, registration number 1117/2020; Greece: Scientific Council of the University Hospital of Ioannina, Protocol Nr 586/6-8-2020; Germany: Ethics Committee Philipps-University Marburg; study file number 163/19; Italy: Comitato Etico per la Sperimentazione Clinica della Provincia di Padova, protocol number CESC 4840/AO/20; Estonia: Reserach Ethics Committee of the University of Tartu (UT REC), protocols 2971T-16 and 327/M-16). The patients/participants provided their written informed consent to participate in this study.

## Author Contributions

MM, GG, and MG wrote the initial draft of the manuscript. MM, GG, AS, SK, CE, PT, SL, PO, KR, KC, AA, BB, and MG contributed to the conception of the intervention and design of the evaluation methodology and contributed to the finalisation of the manuscript and agreed to be accountable for the content of the work. All authors contributed to the article and approved the submitted version.

## Conflict of Interest

The authors declare that the research was conducted in the absence of any commercial or financial relationships that could be construed as a potential conflict of interest. The reviewer MK declared a past co-authorship with one of the authors CE to the handling Editor.

## Publisher's Note

All claims expressed in this article are solely those of the authors and do not necessarily represent those of their affiliated organizations, or those of the publisher, the editors and the reviewers. Any product that may be evaluated in this article, or claim that may be made by its manufacturer, is not guaranteed or endorsed by the publisher.

## Members of the PD_Pal Consortium

Gatsios D, Department of Neurology, Medical School, University of Ioannina, Ioannina, Greece

Fotiadis D, Unit of Medical Technology and Intelligent Information Systems, Department of Material Science and Engineering, University of Ioannina, Ioannina, Greece

Di Marco R, Department of Neuroscience, University of Padua, Padua, Italy

Tedesco C, Department of Neuroscience, University of Padua, Padua, Italy

Krikmann U, Department of Neurology and Neurosurgery, Institute of Clinical Medicine, University of Tartu, Tartu, Estonia

Sikk K, North Estonian Regional Hospital, Tallinn, Estonia

Pedrosa D, Department of Neurology, Philipps University Marburg, Marburg, Germany

Tsakanika A, PD Neurotechnology Ltd, Greece
